# Psychological pathways in enterprise participation in university-industry collaboration: how does social cognitive theory explain participation willingness?

**DOI:** 10.3389/fpsyg.2025.1578950

**Published:** 2025-04-07

**Authors:** Jun Liu

**Affiliations:** Department of Computer, Weifang University of Science and Technology, Weifang, China

**Keywords:** social cognitive theory, university-industry collaboration, enterprise participation, influencing factors, policy empowerment, decision-making mechanisms

## Abstract

**Background:**

With the in-depth implementation of innovation-driven development strategies, University-Industry Collaboration (UIC) has become an important pathway for promoting technological innovation and industrial upgrading. However, enterprises, as one of the main collaboration entities, show significant differences in their participation enthusiasm and depth. Existing research mainly explores UIC influencing factors from resource dependence and knowledge management perspectives, with insufficient exploration of the enterprise cognitive dimension. This study constructs an analytical framework based on social cognitive theory to systematically examine how multiple cognitive factors and environmental factors influence enterprise decision-making in UIC participation.

**Purpose:**

To reveal how observational learning, self-efficacy, outcome expectations, and policy support jointly influence enterprises’ decision-making process in UIC participation, and to explore the moderating role of organizational characteristics in this process.

**Methods:**

Through a questionnaire survey of 300 enterprises in China’s coastal regions, this study employed Structural Equation Modeling (SEM) to analyze the influence mechanisms of observational learning, self-efficacy, outcome expectations, and policy support on enterprises’ willingness to participate in UIC.

**Results:**

The research found that: (1) observational learning, self-efficacy, and outcome expectations have significant positive impacts on enterprise participation willingness (β = 0.285, 0.312, 0.356, *p* < 0.001), with outcome expectations showing the strongest direct effect; (2) risk and cost perceptions significantly inhibit participation willingness (β = −0.245, *p* < 0.001), this indicates enterprises carefully weigh potential benefits against perceived risks when making UIC participation decisions; (3) policy support indirectly promotes participation willingness by enhancing enterprise self-efficacy (β = 0.298, *p* < 0.001); (4) enterprises’ innovation capabilities and resource endowments positively moderate the relationship between policy support and participation willingness (β = 0.187, *p* < 0.01).

**Conclusion:**

This research extends the application of social cognitive theory in inter-organizational collaboration research, provides empirical evidence for policy design and enterprise practice, and emphasizes the need to enhance UIC effectiveness through capacity building, differentiated policy support, and risk management.

## 1 Introduction

With the rapid development of the global economy and technology, University-Industry Collaboration (UIC) has attracted widespread academic attention as an important link connecting higher education and industrial development. By integrating enterprise demands with educational resources, UIC aims to cultivate high-quality talents that meet market needs while promoting industrial transformation and upgrading. However, despite the significant advantages of this collaboration model, enterprise participation enthusiasm and depth show remarkable differences in practice. For example, although the Chinese government actively promotes deep integration between universities and enterprises, many companies still maintain a wait-and-see attitude toward such collaboration, resulting in relatively low overall participation rates (Ministry of Education and Ministry of Industry and Information Technology, 2021). Understanding these participation discrepancies requires examining not only external factors but also the internal cognitive processes that drive enterprise decision-making in UIC contexts.

This study aims to address the following research questions: (1) How do cognitive factors (observational learning, self-efficacy, outcome expectations) and environmental factors (policy support) influence enterprises’ willingness to participate in UIC? (2) What role do organizational characteristics play in moderating these relationships? The rationale for this study lies in bridging the theoretical gap in understanding enterprise decision-making mechanisms within UIC, which has been underexplored in prior research focused on resource dependence and knowledge management. By integrating social cognitive theory, we provide a novel framework to guide policy design and enterprise strategy.

Existing research has explored UIC from multiple dimensions (Tootell et al., 2021; El-Ferik and Al-Naser, 2021; Băban et al., 2022; Arranz et al., 2022). At the motivation level, enterprises participate in UIC mainly to acquire innovation resources and enhance innovation capabilities (Melnychuk et al., 2021), access cutting-edge technologies and talents (Aksoy et al., 2022), reduce R&D costs and disperse innovation risks (Băban and Băban, 2021), thereby strengthening market competitiveness (Cantner et al., 2023). For universities, securing research funding support (Cohen et al., 2024), promoting research achievement transformation (Fernández, 2020), and improving talent cultivation quality (Zhuang and Zhou, 2022) are primary objectives. From a talent cultivation perspective, enhancing graduate employability is a key driving factor for university-enterprise collaboration (Borah et al., 2021), while enterprise participation levels are influenced by multiple factors including internal resource allocation, market environment, and policy support (Smith and Johnson, 2020; Hou et al., 2019).

From a collaboration model perspective, research shows diversification trends. Specifically, collaboration forms include joint R&D, talent cultivation, and joint laboratory construction (Moradi and Noori, 2020; Santos et al., 2021); organizational structures range from loose to tight collaboration, as well as project-based and institutionalized collaboration (Ollila, 2025; Tagliazucchi et al., 2021); operation mechanisms emphasize establishing coordination mechanisms, benefit distribution, and risk-sharing mechanisms (Clauss et al., 2024; O'Dwyer et al., 2022). From a collaboration mechanism perspective, establishing clear communication channels (Pertuz et al., 2021) and cultivating trust and mutual benefit relationships between partners (Figueiredo and Ferreira, 2021) are key elements for successful collaboration.

Factors affecting UIC effectiveness span multiple levels: the organizational level involves cultural differences, management mechanisms, and resource complementarity (Mathisen and Jørgensen, 2021; Cirella and Murphy, 2022); the institutional level includes policy support, incentive mechanisms, and evaluation systems (Li et al., 2024; Yao et al., 2021); the relationship level emphasizes trust, communication mechanisms, and collaboration experience (Tootell et al., 2021; El-Ferik and Al-Naser, 2021). From a regional development perspective, collaboration models need to be adjusted according to local economic characteristics (Chen and Xu, 2017), while balancing high collaboration costs and uncertainties (Neri et al., 2021). Research shows that organizational culture atmosphere and professional commitment have significant positive effects on enterprise participation behavior (Benson and Chau, 2022). In performance manifestation, UIC shows positive effects in innovation output (patent output, technological breakthroughs, and knowledge spillovers) (Yin et al., 2023; Goel, 2021), economic benefits (revenue growth, cost savings, and market value enhancement) (Cohen et al., 2024; Bamford et al., 2023), and talent cultivation (student employment quality and entrepreneurial ability development) (Shenkoya et al., 2023; Tinnakorn et al., 2021).

However, existing research has the following limitations: First, research primarily focuses on enterprises in developed countries, with insufficient attention to those in developing countries like China (Yu, 2021); Second, most adopt qualitative research methods, lacking large-scale empirical validation; Third, although policy support is widely considered a key factor in promoting UIC, its action mechanism has not been deeply revealed (Li et al., 2024; Yao et al., 2021), with insufficient attention to the micro-level internal mechanisms of enterprise participation behavior. Additionally, research mainly relies on traditional perspectives such as resource dependence theory and knowledge management theory (Tootell et al., 2021; Corsi et al., 2020), lacking in-depth analysis of enterprise cognition and decision-making processes. From a policy support perspective, insufficient resource input and inadequate incentive measures are the main factors constraining collaboration effectiveness (Qin and Lei, 2024). In response to these issues, research suggests enhancing synergistic effects through integrating educational and industrial resources and constructing long-term collaboration mechanisms (Zhang and Perey, 2024).

Social cognitive theory provides an effective theoretical framework for understanding enterprise participation in UIC. This theory emphasizes that individual or organizational behavior is influenced not only by internal attitudes and subjective norms but also by multiple factors such as observational learning, self-efficacy, and outcome expectations (Bandura, 1986). Research shows that self-efficacy plays a particularly important role in education and organizational behavior, especially in highly uncertain environments (Schunk, 1991). Enterprises can form positive outcome expectations by observing successful collaboration cases of others (Liu, 2016), while policy environment plays a key regulatory role in enterprise participation behavior (Chen et al., 2022). Although existing research emphasizes the promoting role of policy support for UIC, some studies point out that excessive reliance on policy subsidies may lead enterprises to form “path dependence,” weakening their endogenous collaboration motivation. This research reveals how policy support can avoid this trap by introducing the role of self-efficacy, namely by stimulating enterprise initiative through capability building rather than mere resource input.

Despite the widely recognized strategic value of UIC, the heterogeneity in enterprise participation motivation has not been fully explained. Existing theories mostly focus on resource exchange but neglect the cognitive black box of enterprise decision-making. This research attempts to fill these theoretical gaps. By introducing a social cognitive theory perspective, this study constructs an integrated analytical framework to systematically examine how cognitive factors (observational learning, self-efficacy, outcome expectations) and environmental factors (policy support) jointly influence enterprises’ decision-making process in UIC participation. Special attention is paid to the intermediary mechanism through which policy support influences participation willingness by enhancing enterprise self-efficacy, as well as the moderating role of organizational characteristics, providing a new theoretical perspective for understanding the transmission mechanism of policy effects.

The structure of this research is as follows: Section 2 constructs the theoretical framework, Section 3 explains the research design, Section 4 presents the empirical research results, and the final section summarizes the research findings and discusses theoretical and practical implications.

## 2 Theoretical framework and research hypotheses

### 2.1 Theoretical framework

Social cognitive theory provides an effective theoretical framework for understanding enterprise participation in UIC behavior. This theory, proposed by Bandura (1986), emphasizes that individual (or organizational) behavior is influenced by the mutual interaction of cognitive factors, environmental factors, and behavioral outcomes. This theory emphasizes the core role of cognitive evaluation in behavioral decision-making, particularly suitable for explaining enterprise strategic choices in uncertain environments.

**Figure 1:**
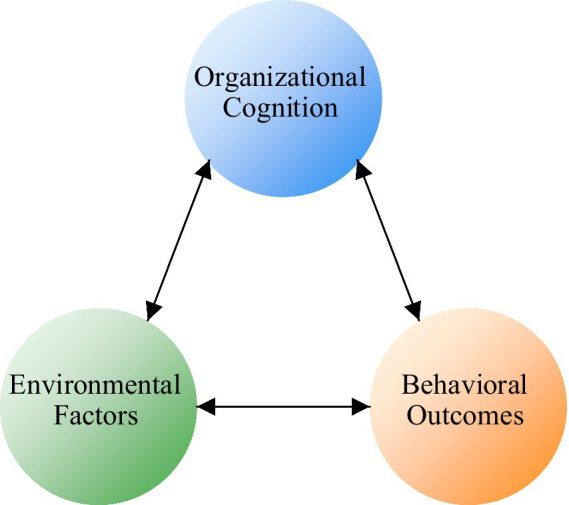
Triadic reciprocal model of social cognitive theory applied to UIC context.

In the triadic reciprocal model of social cognitive theory (Figure 1), cognitive elements (such as judgments and expectations), environmental elements (such as norms and resources), and behavioral outcomes form a continuous cycle of mutual influence. Specifically in the UIC context, this interactive relationship manifests as: enterprise self-efficacy cognition (confidence in managing and executing collaboration projects) influences its participation decisions, while collaboration experience strengthens or adjusts this cognitive judgment; policy environment (such as innovation incentives) both shapes enterprise efficacy perception and directly affects the specific implementation of collaboration behavior; enterprise participation behavior (such as R&D investment) may bring technological breakthroughs, thereby changing the innovation environment and policy orientation. The triadic reciprocal model of social cognitive theory emphasizes that enterprise decisions to participate in UIC result from the dynamic interaction of cognitive factors (such as self-efficacy), behavioral outcomes (such as expected benefits), and environmental support (such as policies). This interactive mechanism in the UIC context manifests as: enterprises form cognitive templates through observational learning, combined with self-efficacy appraisal and external policy incentives (environmental scaffolding), ultimately driving collaborative behavior.

In this study, “policy support” is defined as government-driven initiatives (e.g., subsidies, tax incentives, and regulatory frameworks) perceived by enterprises, distinct from university-specific policies. “Organizational characteristics” are operationalized into two dimensions: (1) innovation capability (e.g., R&D investment, innovation management systems) and (2) resource endowment (e.g., financial strength, talent quality), consistent with prior studies (Mathisen and Jørgensen, 2021; Cirella and Murphy, 2022).

Recent studies have advanced our understanding of policy-environment interactions in UIC. For example, Li et al. (2024) explored how environmental regulations shape collaborative innovation in green technologies but did not address the role of self-efficacy. Similarly, Yao et al. (2021) emphasized the evolution of China’s industry-university-research collaboration but focused on macro-level trends rather than cognitive mechanisms. Our study uniquely integrates social cognitive theory with institutional perspectives to reveal how policy support enhances self-efficacy, thereby fostering participation willingness—a pathway not previously examined. Furthermore, we extend Tootell et al.’s (2021) work on observational learning by demonstrating its applicability in developing economies like China, where institutional contexts differ markedly from Western settings. This dual focus on cognitive and environmental factors distinguishes our research from prior work centered on resource exchange or knowledge transfer (Tootell et al., 2021; Corsi et al., 2020).

By incorporating enterprise cognition, policy environment, and behavioral outcomes into a unified analytical framework, this research not only embodies social cognitive theory’s deep insight into behavioral formation mechanisms but also reflects the specificity of enterprise decision-making in UIC contexts. This integrated framework helps to more comprehensively understand the multiple factors affecting enterprise participation in UIC and their interactions, laying a theoretical foundation for subsequent empirical analysis.

First, social cognitive theory emphasizes the key role of observational learning in behavior formation. Liu’s (2016) research found that organizations can form positive behavioral tendencies by observing and learning from other organizations’ successful experiences. Tootell et al. (2021) and Pertuz et al. (2021) further verified that in inter-organizational collaboration contexts, demonstration effects significantly promote organizational participation willingness. This observational learning mechanism enables enterprises to acquire knowledge from others’ experiences, reducing decision-making uncertainty.

Second, organizational cognitive factors, especially self-efficacy, play an important role in the decision-making process. Schunk (1991) research shows that self-efficacy is a key factor in predicting organizational behavior, particularly in highly uncertain environments. Chen et al. (2022) empirically verified that in UIC contexts, high self-efficacy significantly enhances enterprise participation willingness. This finding is also supported by Băban et al.’s (2022) research, which emphasizes that self-efficacy is a core psychological mechanism driving inter-organizational collaboration.

Third, outcome expectations have important influences on organizational decision-making. Melnychuk et al. (2021) and Cantner et al. (2023) found that enterprises’ evaluation of expected collaboration benefits directly affects their participation decisions. Particularly in UIC contexts, expected innovation performance and knowledge spillover effects are key factors driving enterprise participation, as further verified in recent research by Yin et al. (2023) and Goel (2021).

However, enterprises also consider potential risks and costs when evaluating participation decisions. Băban and Băban’s (2021) research revealed the inhibitory effect of risk perception on organizational collaboration behavior. Neri et al. (2021), through empirical research on European enterprises, confirmed that cost and risk factors are important barriers to inter-organizational collaboration. This trade-off mechanism makes enterprises more cautious in decision-making.

Additionally, environmental support, especially policy support, plays a key role in promoting enterprise participation. Li et al.’s (2024) research shows that institutional environment has a significant regulatory effect on organizational behavior. Yao et al. (2021) further found that policy support effectively promotes deep university-enterprise collaboration by reducing collaboration barriers and optimizing expected returns. This support mechanism not only directly affects enterprise decisions but also indirectly functions by enhancing organizational self-efficacy.

Finally, organizational characteristics, such as innovation capability and resource endowment, play an important moderating role in UIC participation processes. Mathisen and Jørgensen’s (2021) research emphasizes the importance of organizational characteristics in knowledge collaboration. Cirella and Murphy’s (2022) and Benson and Chau’s (2022) research further verified that organizational innovation orientation and resource capabilities significantly affect their performance and benefit acquisition ability in collaboration processes. Although policy support is generally viewed as a UIC catalyst, its effect may vary due to enterprise heterogeneity. For example, Hou et al. (2019) found that excessive subsidies could weaken small and medium enterprises’ autonomy, while Yao et al. (2021) emphasize that policies need to be coordinated with market mechanisms. This research provides a new interpretative framework for this controversy by introducing the moderating effect of organizational characteristics.

### 2.2 Research hypotheses

Based on the above theoretical framework, this research proposes the following hypotheses:

*H1*: Enterprises can significantly increase their willingness to participate in UIC by observing other enterprises' successful collaboration experiences.

*H2*: The higher an enterprise's self-efficacy, the more likely it is to participate in UIC.

*H3*: Enterprises' positive outcome expectations regarding UIC show a significant positive correlation with their participation willingness.

*H4*: Perceived collaboration risks and costs will significantly reduce enterprises' willingness to participate in UIC.

*H5*: Policy support indirectly increases enterprises' willingness to participate in UIC by enhancing their self-efficacy.

*H6*: Enterprises' innovation capabilities and resource endowments moderate the impact of environmental factors on their willingness to participate in UIC.

**Figure 2:**
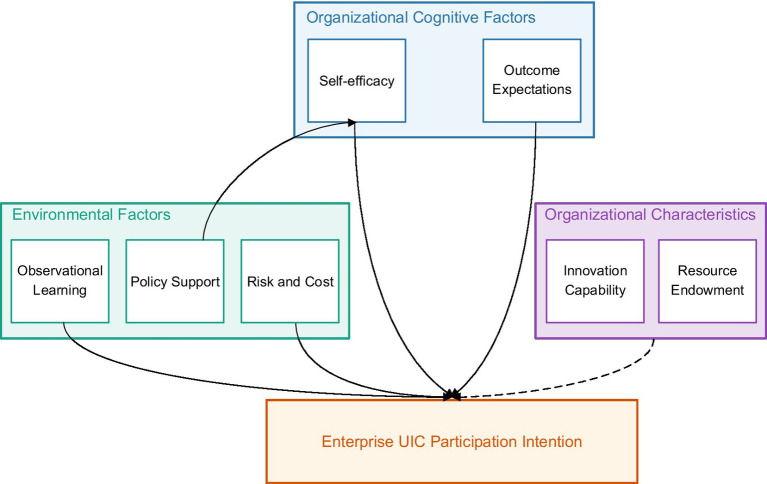
Conceptual model of the mechanisms influencing enterprise participation in UIC.

Based on the above theoretical framework, the conceptual model proposed in this research is shown in Figure 1. This model shows the hypothesized relationships between key constructs and their expected impact directions. Figure 2 intuitively presents how organizational cognitive factors (self-efficacy and outcome expectations), environmental factors (observational learning and policy support) influence enterprise willingness to participate in UIC, the inhibitory effect of risk and cost perception, and the moderating role of organizational characteristics. Solid lines indicate direct effects (including positive and negative impacts), dashed lines indicate moderating effects, and arrows with H1-H6 represent corresponding research hypotheses. This integrated model not only embodies the complex interactive relationships between variables but also provides theoretical guidance for subsequent empirical analysis.

This model unfolds from three dimensions: First, at the cognitive level, it examines the impact of enterprise self-efficacy and outcome expectations on participation willingness; Second, at the environmental level, it analyzes the action mechanisms of observational learning and policy support; Finally, at the behavioral level, it explores the inhibitory effect of risk and cost perception. Meanwhile, the research also focuses on the moderating role of enterprise characteristics (innovation capability and resource endowment), revealing the differentiated responses of different types of enterprises to external support. This research incorporates risk and cost perception into environmental factors because this perception essentially reflects enterprise evaluation of its operating environment. In the UIC context, enterprise risk-cost judgment is primarily based on understanding the external environment, including perception of intellectual property protection, collaboration norms, and market uncertainties.

## 3 Research design

**Figure 3:**
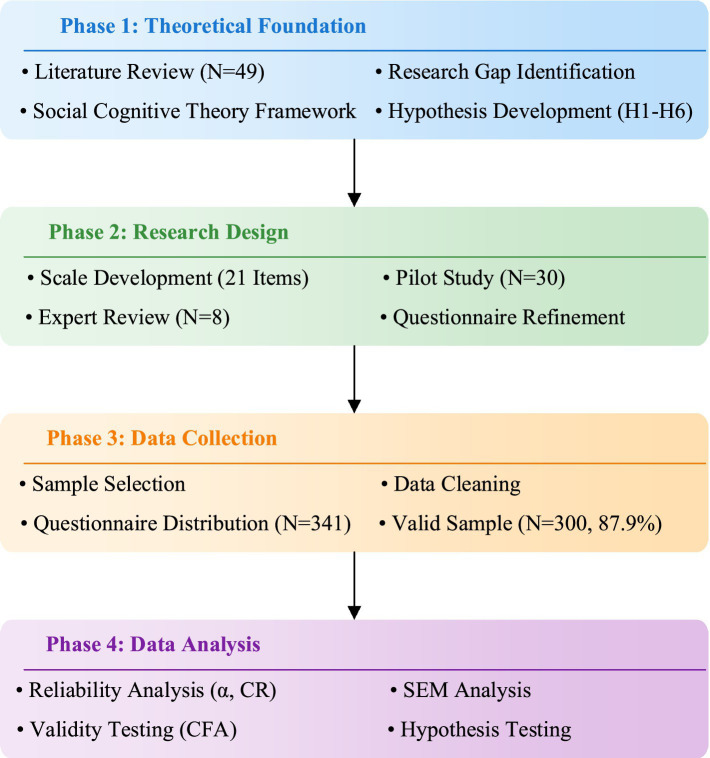
The overall design framework of this research.

This research employed questionnaire survey methods to collect data. To ensure the reliability and validity of research results, strict control measures were adopted in questionnaire design, sample selection, and data collection. To ensure the scientific nature and standardization of the research, this study adopted a systematic research design process. Figure 3 shows the overall design framework of this research. The research is divided into four main phases: theoretical foundation (Phase 1), research design (Phase 2), data collection (Phase 3), and data analysis (Phase 4). In the theoretical foundation phase, a theoretical framework was constructed and research hypotheses proposed through literature review (*N* = 49); the research design phase developed a scale containing 21 items, refined after expert review (*N* = 8) and pre-testing (*N* = 30); the data collection phase distributed 341 questionnaires, obtaining 300 valid samples, with an effective rate of 87.9%; the data analysis phase employed structural equation modeling for hypothesis testing, ensuring data quality through reliability and validity analysis. This systematic research design provided important assurance for the reliability of the research results.

### 3.1 Research subjects

This research targeted enterprises from different regions of China that participate in or intend to participate in UIC, effectively capturing the forefront dynamics of UIC practices. A stratified sampling method was employed, with quota sampling based on enterprise size, ownership type, and industry background to ensure sample representativeness. Data collection work was conducted between January and June 2024, combining face-to-face interviews and online questionnaires. As shown in Table 1, among the sample enterprises, manufacturing accounted for 41.3% (124 enterprises), followed by healthcare industry 18.7% (56 enterprises) and automotive industry 12.7% (38 enterprises). In terms of enterprise size, enterprises with fewer than 100 employees accounted for 51.3% (154 enterprises), while large enterprises with over 1,000 employees accounted for 18.0% (54 enterprises). Regarding enterprise nature, private enterprises accounted for 43.3% (130 enterprises), followed by sole proprietorships 19.3% (58 enterprises) and state-owned enterprises 15.3% (46 enterprises).

**Table 1:** Descriptive statistics of the sample.

Question	Options	Frequency (*N* = 300)
Enterprise registration duration (Q1)	<5 years	82
	5–9 years	39
	10–20 years	115
	>20 years	64
Employee size (Q2)	<100 employees	154
	100–199 employees	38
	200–499 employees	33
	500–1,000 employees	21
	>1,000 employees	54
Ownership type (Q3)	Private enterprise	130
	Sole proprietorship	58
	State-owned enterprise	46
	Foreign-funded enterprise	26
	Hong Kong, Macau, and Taiwan invested enterprise	17
	Other	23
Industry sector (Q4)	Manufacturing	124
	Healthcare	56
	Automotive industry	38
	Commerce	22
	Construction	21
	Information technology	12
	Other	27

To ensure data quality, this research implemented strict control measures: First, questionnaire respondents were required to be enterprise senior managers or department heads responsible for UIC decisions, ensuring respondents thoroughly understood enterprise strategic decisions and collaboration willingness; Second, questionnaire distribution and completion processes were standardized, with mandatory response and logical jump functions set in online questionnaires to ensure completion integrity; Third, reverse items and logical verification items were set to identify and eliminate invalid responses. As shown in Table 1, after eliminating 41 invalid questionnaires from the total 341 distributed, 300 valid questionnaires were ultimately obtained, with an effective recovery rate of 87.9%.

Questionnaires were completed by senior managers (e.g., CEOs, R&D directors) or department heads directly responsible for UIC decisions, ensuring alignment with organizational strategic priorities. This approach aligns with prior studies (Smith and Johnson, 2020; O'Dwyer et al., 2022), which confirm that such respondents reliably represent enterprise intent in inter-organizational collaboration contexts.

### 3.2 Variable measurement

The development of measurement scales followed the scale development procedure proposed by Likert (1932), including literature review, scale design, expert review, and pre-testing four phases (Harpe, 2015).

The scale development involved four phases: (1) Literature review identified initial items; (2) Expert review by 5 industry managers (e.g., R&D directors) and 3 academics refined wording; (3) Pre-testing with 30 enterprises assessed clarity and relevance; (4) Final adjustments ensured alignment with theoretical constructs. Cronbach’s α for all constructs exceeded 0.8, confirming reliability (Likert, 1932; Harpe, 2015). For example, the self-efficacy scale included items such as “Our enterprise can effectively manage UIC projects,” validated through iterative feedback.

In the first phase, initial scales were constructed based on existing literature; in the second phase, 5 enterprise managers (including 2 general managers, 2 R&D directors, and 1 UIC department head) and 3 management professors were invited for expert review, with item expressions modified; in the third phase, 30 enterprises of different sizes and industries were selected for pre-testing, with feedback collected and scales modified; in the fourth phase, following Joshi et al.’s (2015) suggestion, all constructs were measured using five-point Likert scales (1 = “strongly disagree,” 5 = “strongly agree”).

This research employed multi-item scales to measure latent variables. The observational learning dimension mainly measured the extent to which enterprises pay attention to and learn from other successful UIC cases, including 3 measurement items: “Our enterprise closely follows other enterprises’ performance in UIC,” “Our enterprise gains valuable insights from other enterprises’ UIC experiences,” and “Our enterprise actively learns from successful UIC practice cases.” The self-efficacy dimension evaluated enterprises’ confidence in their ability to conduct UIC, measured through 3 items: “Our enterprise can effectively manage UIC projects,” “Our enterprise possesses various capabilities needed for UIC,” and “Our enterprise is confident in handling various challenges in the UIC process.”

The outcome expectations dimension measured enterprises’ evaluation of potential benefits from UIC, containing 3 measurement items: “Participating in UIC helps acquire key technologies and talents,” “Participating in UIC can enhance enterprise market competitiveness,” and “Participating in UIC can promote enterprise innovation development.” The risk and cost dimension assessed enterprises’ perception of potential risks and required investment in UIC, measured through 3 items: “UIC may bring intellectual property dispute risks,” “UIC requires investment of substantial resources and costs,” and “UIC may lead to core technology leakage.”

The policy support dimension measured the degree of external support perceived by enterprises, including 3 measurement items: “Local government provides clear policy support for UIC,” “Existing policies can effectively reduce risks for enterprises participating in UIC,” and “Government provides sufficient resource support for UIC.” Organizational characteristics included innovation capability and resource endowment sub-dimensions. Innovation capability was measured through 3 items: “The enterprise has strong R&D innovation capabilities,” “The enterprise values technological innovation investment,” and “The enterprise has a good innovation management system.” Resource endowment similarly contained 3 measurement items: “The enterprise has sufficient R&D resources,” “The enterprise has strong financial strength,” and “The enterprise has a high-quality talent team.”

Enterprise participation in UIC willingness as the dependent variable mainly measured enterprise attitudes and investment intentions toward UIC, including 3 measurement items: “The enterprise plans to increase UIC investment,” “The enterprise is willing to participate in UIC long-term,” and “The enterprise values cooperation development with universities.” All constructs were measured using five-point Likert scales (1 = “strongly disagree,” 5 = “strongly agree”).

### 3.3 Data analysis methods

This research employed Structural Equation Modeling (SEM) for hypothesis testing. The choice of SEM was based on three considerations: First, SEM can simultaneously process measurement models and structural models, particularly suitable for testing the complex causal relationships proposed in this research; Second, SEM can evaluate direct and indirect effects between latent variables, helping reveal the intermediary mechanism through which policy support influences participation willingness via self-efficacy; Third, SEM can effectively handle moderation effect analysis, suitable for testing the moderating role of organizational characteristics.

The data analysis process was divided into four steps: First, using SPSS 26.0 for descriptive statistics and correlation analysis to preliminarily understand relationship patterns between variables; Second, using AMOS 26.0 for Confirmatory Factor Analysis (CFA) to evaluate measurement model reliability (including Cronbach’s α coefficient and composite reliability CR) and validity (including convergent validity and discriminant validity); Third, constructing a structural equation model, employing maximum likelihood method to estimate path coefficients and test direct effect hypotheses; Fourth, using Bootstrap method (5,000 resamples) to test the significance of mediating effects, and using multi-group analysis methods to test the moderating effects of organizational characteristics. In methodology, we employed the percentile bootstrap method with 5,000 resamples to test the significance of the mediating effect, reporting 95% confidence intervals to ensure robust statistical inference.

## 4 Empirical study and results analysis

### 4.1 Reliability and validity testing

The reliability and validity test results for latent variables are shown in Table 2. The observational learning dimension had a Cronbach’s α of 0.893 and CR value of 0.901; the self-efficacy dimension had a Cronbach’s α of 0.876 and CR value of 0.885; the outcome expectations dimension had a Cronbach’s α of 0.902 and CR value of 0.911; the risk and cost dimension had a Cronbach’s α of 0.867 and CR value of 0.879; the policy support dimension had a Cronbach’s α of 0.883 and CR value of 0.892; the organizational characteristics dimension had a Cronbach’s α of 0.895 and CR value of 0.904; the enterprise participation willingness dimension had a Cronbach’s α of 0.888 and CR value of 0.897. Analysis results show that standardized loadings of all observed variables on their respective latent variables were between 0.812 and 0.891, significantly higher than the recommended threshold of 0.7. Composite reliability (CR) values for each latent variable ranged from 0.879 to 0.911, and average variance extracted (AVE) values ranged from 0.627 to 0.798, all meeting convergent validity requirements.

**Table 2:** Reliability and convergent validity test results for questionnaire data.

Latent variable	Item	Standardized loading	Cronbach’s α	CR	AVE
Observational learning	OL1	0.845	0.893	0.901	0.743
	OL2	0.878			
	OL3	0.876			
Self-efficacy	SE1	0.856	0.876	0.885	0.721
	SE2	0.861			
	SE3	0.842			
Outcome expectations	EX1	0.867	0.902	0.911	0.798
	EX2	0.883			
	EX3	0.891			
Risk and cost	RC1	0.812	0.867	0.879	0.684
	RC2	0.831			
	RC3	0.835			
Policy support	PS1	0.823	0.883	0.892	0.727
	PS2	0.832			
	PS3	0.856			
Organizational characteristics	OC1	0.834	0.895	0.904	0.759
	OC2	0.839			
	OC3	0.867			
Participation intention	PI1	0.825	0.888	0.897	0.627
	PI2	0.868			
	PI3	0.848			

All scales had Cronbach’s α > 0.8, composite reliability CR > 0.7, AVE > 0.5, meeting convergent validity requirements.

To verify the structural validity of the measurement model, this research conducted confirmatory factor analysis, with results shown in Figure 3. Analysis results indicate that the measurement model has good fit (χ^2^/df = 2.245, RMSEA = 0.064, CFI = 0.942, TLI = 0.935). Correlation analysis between latent variables shows that self-efficacy and outcome expectations (*r* = 0.445, *p* < 0.001), observational learning and self-efficacy (*r* = 0.412, *p* < 0.001) display significant positive correlations, while risk and cost with outcome expectations (*r* = −0.312, *p* < 0.001) and self-efficacy (*r* = −0.298, *p* < 0.001) show significant negative correlations. These moderate correlation coefficients indicate that latent variables both relate to each other and maintain good discrimination. The positive correlation between policy support and self-efficacy (*r* = 0.423, *p* < 0.001) provides a foundation for subsequent mediating effect analysis.

**Figure 4:**
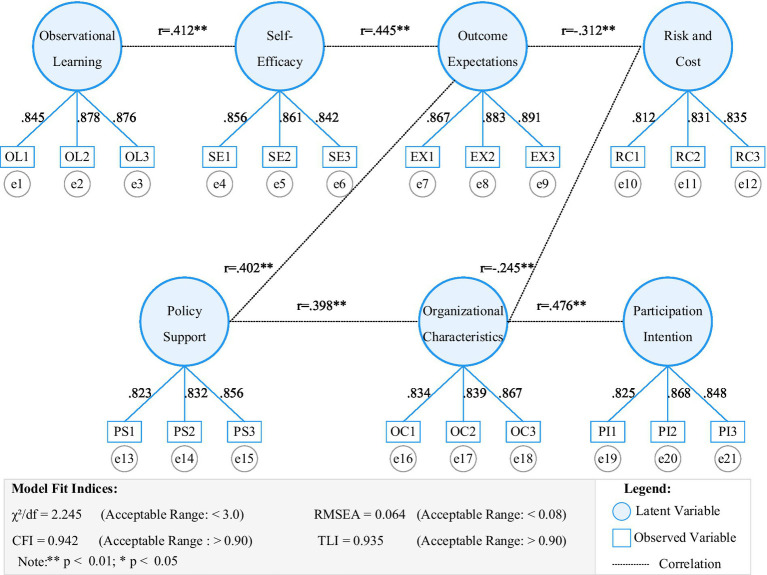
Measurement model validation.

Figure 4 intuitively displays the structural relationships and main parameter estimation results of the measurement model, including standardized path coefficients, error terms, and correlation coefficients between latent variables. The model’s overall fit indices are good (χ^2^/df = 2.245, RMSEA = 0.064, CFI = 0.942, TLI = 0.935), indicating the measurement model has good construct validity.

### 4.2 Correlation analysis

Table 3 presents the correlation coefficient matrix between latent variables. Results show that except for the risk and cost dimension, other independent variables all show significant positive correlations with enterprise participation willingness. Specifically, the correlation coefficient between observational learning and participation willingness is 0.452 (*p* < 0.01), between self-efficacy and participation willingness is 0.487 (*p* < 0.01), between outcome expectations and participation willingness is 0.523 (*p* < 0.01), between policy support and participation willingness is 0.445 (*p* < 0.01), and between organizational characteristics and participation willingness is 0.476 (*p* < 0.01). The risk and cost dimension shows a significant negative correlation with participation willingness, with a correlation coefficient of −0.389 (*p* < 0.01). These preliminary results provide support for subsequent hypothesis testing.

**Table 3:** Correlation matrix of variables.

Variables	1	2	3	4	5	6	7
Observational learning	1						
Self-efficacy	0.412**	1					
Outcome expectations	0.389**	0.445**	1				
Risk and cost	−0.256**	−0.298**	−0.312**	1			
Policy support	0.378**	0.423**	0.402**	−0.287**	1		
Organizational characteristics	0.401**	0.467**	0.434**	−0.245**	0.398**	1	
Participation intention	0.452**	0.487**	0.523**	−0.389**	0.445**	0.476**	1

**p < 0.01, *p < 0.05.

### 4.3 Hypothesis testing

This research employed structural equation modeling (SEM) to test research hypotheses. Model fit was evaluated, with results showing: χ^2^/df = 2.245 (less than 3), RMSEA = 0.064 (less than 0.08), CFI = 0.942, TLI = 0.935, IFI = 0.944 (all greater than 0.9), indicating the model has good fit. Table 4 presents the main effect hypothesis testing results.

**Table 4:** Results of hypothesis testing.

Path	Standardized path coefficient	*t*-value	*p*-value	Result
H1: Observational learning → participation intention	0.285	4.567	<0.001	Supported
H2: Self-efficacy → participation intention	0.312	5.234	<0.001	Supported
H3: Outcome expectations → participation intention	0.356	5.789	<0.001	Supported
H4: Risk and cost → participation intention	−0.245	−4.123	<0.001	Supported
H5: Policy support → self-efficacy	0.298	4.876	<0.001	Supported
H6: Moderating effect of organizational characteristics	0.187	3.234	<0.01	Supported

Research results show that: (1) observational learning has a significant positive impact on enterprise participation willingness (β = 0.285, *p* < 0.001), supporting H1; (2) self-efficacy has a significant positive impact on participation willingness (β = 0.312, *p* < 0.001), supporting H2; (3) outcome expectations have a significant positive impact on participation willingness (β = 0.356, *p* < 0.001), supporting H3; (4) risk and cost have a significant negative impact on participation willingness (β = −0.245, *p* < 0.001), supporting H4; (5) policy support indirectly influences participation willingness by enhancing self-efficacy (β = 0.298, *p* < 0.001), supporting H5. This mediating effect indicates that policy support not only directly lowers enterprise participation thresholds but more importantly stimulates their participation enthusiasm by enhancing their capability perception. To verify the robustness of the mediating effect, this research employed the Bootstrap method (5,000 resamples), with results showing the 95% confidence interval for the mediating effect is [0.156, 0.387], not including 0, further confirming the mediating effect’s significance. The mediating effect of policy support through self-efficacy accounts for 42% of the total effect (indirect effect = 0.125, direct effect = 0.173), indicating its function is realized more through empowerment rather than direct incentives. Taking a biopharmaceutical enterprise as an example, UIC management training provided by the government (policy support) significantly enhanced its project management confidence (self-efficacy), thereby promoting its joint laboratory establishment with universities.

**Figure 5:**
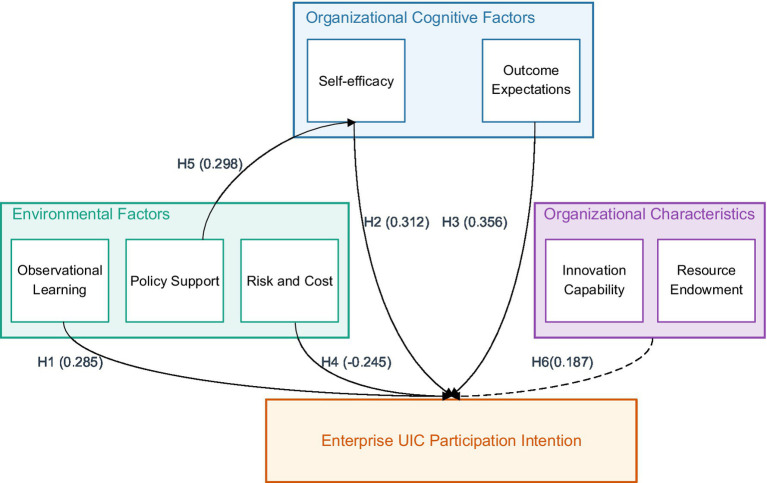
Results of SEM analysis.

Through structural equation modeling to test research hypotheses, the complex influence mechanisms of enterprise participation in UIC were revealed, with analysis results shown in Figure 4. The model’s overall fit is good (χ^2^/df = 2.245, RMSEA = 0.064, CFI = 0.942, TLI = 0.935). From path analysis results, observational learning (β = 0.285, *p* < 0.001), self-efficacy (β = 0.312, *p* < 0.001), and outcome expectations (β = 0.356, *p* < 0.001) all produce significant positive impacts on enterprise participation willingness, while risk and cost perception shows a significant negative impact (β = −0.245, *p* < 0.001). Policy support indirectly influences participation willingness through self-efficacy (β = 0.298, *p* < 0.001), while the moderating effect of organizational characteristics is also verified (β = 0.187, *p* < 0.01). Figure 5 intuitively displays the action paths and effect sizes between variables, with different paths and arrows representing direct effects, indirect effects, and moderating effects, and numerical annotations reflecting relationship strength and significance levels. These findings support the research hypotheses. Notably, outcome expectations show the most significant impact, indicating that enterprises highly value potential benefits from participating in UIC during the decision-making process.

### 4.4 Moderating effect

To test the moderating role of organizational characteristics (H6), this research employed a multi-group analysis method. First, the sample was divided into high-score group (*n* = 152) and low-score group (*n* = 148) based on the median (3.45) of organizational characteristics scores.

This median-split approach follows established practices in moderation analysis to compare high and low groups effectively, as recommended by Iacobucci et al. (2015). First, the sample was divided into high-score group (*n* = 152) and low-score group (*n* = 148) based on the median (3.45) of organizational characteristics scores. This median-split approach follows established practices in moderation analysis to compare high and low groups effectively, as recommended by Iacobucci et al. (2015).

As shown in Table 5, the impact of policy support on participation willingness in the high-score group (β = 0.412, *p* < 0.001) was significantly stronger than in the low-score group (β = 0.225, *p* < 0.01). Further chi-square difference test results (Δχ^2^ = 8.567, *p* < 0.01) supported the existence of the moderating effect. Results show that organizational characteristics play a significant moderating role in the process of policy support influencing enterprise participation willingness. Specifically, for enterprises with higher organizational characteristic scores, policy support has a stronger impact on participation willingness (β = 0.187, *p* < 0.01).

**Table 5:** Analysis of moderating effects of organizational characteristics.

Path relationship	High group (*n* = 152)	Low group (*n* = 148)	Difference test
Policy support → participation intention	β = 0.412*** (*t* = 5.678)	β = 0.225** (*t* = 3.234)	Δχ^2^ = 8.567**
Self-efficacy → participation intention	β = 0.389*** (*t* = 5.123)	β = 0.234** (*t* = 3.456)	Δχ^2^ = 7.234**
Observational learning → participation intention	β = 0.356*** (*t* = 4.789)	β = 0.213** (*t* = 3.123)	Δχ^2^ = 6.789**

***p < 0.001, **p < 0.01, *p < 0.05.

**Figure 6:**
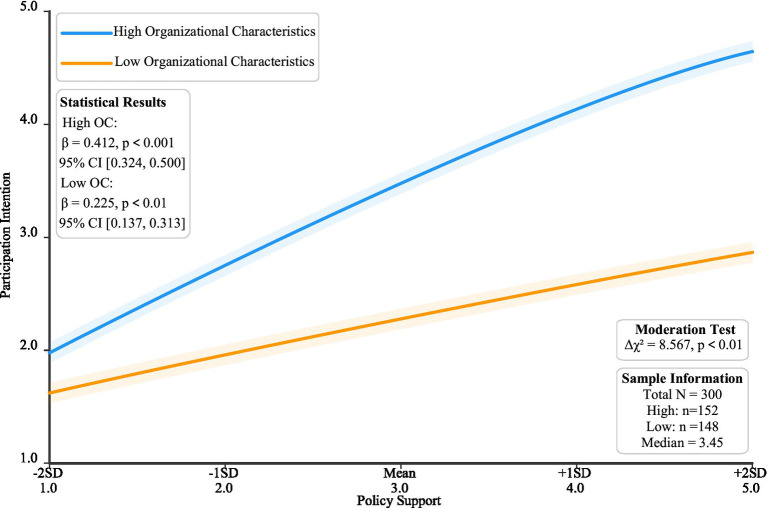
Moderating effect of organizational characteristics.

To visually demonstrate this moderating effect, Figure 6 presents the relationship between policy support and participation willingness at different organizational characteristic levels. As shown in Figure 6, under policy support at different standard deviation levels, the two groups of enterprises show significantly different participation willingness change trends. Specifically, the high organizational characteristics group shows stronger policy responsiveness (β = 0.412, *p* < 0.001, 95% CI [0.324, 0.500]), with participation willingness significantly increasing from 2.1 at -2SD policy support to 4.9 at +2SD. In contrast, the low organizational characteristics group shows relatively weaker policy responsiveness (β = 0.225, *p* < 0.01, 95% CI [0.137, 0.313]), with a smaller increase in participation willingness (from 1.8 to 3.4). The significant difference in slopes between the two groups (Δχ^2^ = 8.567, *p* < 0.01) further confirms the moderating role of organizational characteristics. The confidence interval bands in the figure indicate that this moderating effect is statistically robust, supporting hypothesis H6. This finding implies that enterprises with higher organizational characteristic levels can better utilize policy support, showing stronger participation motivation.

### 4.5 Robustness testing

To ensure the reliability of research results, this research conducted a series of robustness tests. First, different sample grouping methods (such as industry type, enterprise size) were used to repeat the analysis, with results showing core findings remained stable. Second, the stability of the model was tested through stepwise introduction of control variables, with results showing the direction and significance levels of main variables remained largely unchanged. Finally, alternative variables (such as using R&D investment intensity to replace innovation capability) were used for testing, with results still supporting the main conclusions of this research. To exclude potential endogeneity interference, this research employed Two-Stage Least Squares (2SLS), using “industry average policy support level” as an instrumental variable, with results showing no substantial changes in core variable coefficient direction and significance (β = 0.327, *p* < 0.001) (Table 6).

**Table 6:** Robustness tests of model with different control variables.

Model	χ^2^/df	RMSEA	CFI	TLI	IFI
Base model	2.245	0.064	0.942	0.935	0.944
Adding size	2.267	0.066	0.94	0.933	0.941
Adding duration	2.289	0.067	0.938	0.931	0.939
Full control	2.312	0.069	0.936	0.929	0.937

Acceptable ranges: χ^2^/df < 3.0; RMSEA < 0.08; CFI, TLI, IFI > 0.90.

### 4.6 Analysis of control variable effects

This research also analyzed the impact of control variables. Results show that enterprise size is significantly positively correlated with participation willingness (β = 0.156, *p* < 0.05), indicating that large enterprises are more inclined to participate in UIC. The impact of enterprise duration is not significant (β = 0.078, *p* > 0.05), suggesting that enterprise development stage is not a key factor determining its participation in UIC. The impact of ownership type varies, with foreign-funded enterprises (β = 0.189, *p* < 0.01) and state-owned enterprises (β = 0.167, *p* < 0.01) showing significantly higher participation willingness than other types of enterprises. These findings provide a more comprehensive perspective for understanding factors influencing enterprise participation in UIC. This complex influence mechanism indicates that enterprise decision-making in UIC participation is jointly influenced by multiple factors, requiring coordinated advancement from multiple levels to enhance enterprise participation enthusiasm and effectiveness.

## 5 Research discussion and implications

### 5.1 Research findings

Based on social cognitive theory, this research reveals a series of important findings by constructing and validating an influence mechanism model of enterprise participation in UIC. Research results show that observational learning, as an important pathway for enterprise cognitive formation, significantly enhances enterprise willingness to participate in UIC by reducing decision-making uncertainty (β = 0.285, *p* < 0.001). This learning effect not only provides enterprises with practical experiences for reference but also forms a positive demonstration effect. This finding deepens Liu’s (2016) research on inter-organizational learning mechanisms and confirms Tootell et al.’s (2021) discussion on the importance of demonstration effects in organizational decision-making.

Self-efficacy plays a core role in enterprise participation in UIC processes (β = 0.312, *p* < 0.001). Enterprises’ evaluation of their ability to manage and execute UIC projects directly influences their participation decisions, with this impact being particularly significant in highly uncertain collaboration environments. This finding resonates with Chen et al.’s (2022) research and deepens Băban et al.’s (2022) theoretical views on organizational self-efficacy’s role in cross-organizational collaboration. Particularly in complex collaboration contexts, self-efficacy not only affects enterprises’ initial participation decisions but also influences their subsequent investment levels and sustained commitment.

The standardized path coefficient of outcome expectations is significantly higher than other variables (β = 0.356 vs. observational learning β = 0.285; self-efficacy β = 0.312), indicating that rational evaluation of collaboration benefits is the core driver of enterprise decision-making, reflecting enterprises’ rational assessment of potential benefits from UIC. This finding supports Melnychuk et al.’s (2021) argument about the leading position of expected benefits in organizational decision-making, while also confirming Cantner et al. (2023) research findings on innovation collaboration motivations. Through in-depth analysis, it was found that enterprises particularly focus on UIC’s potential contributions in acquiring innovation resources, enhancing innovation capabilities, and strengthening market competitiveness.

Risk and cost perception produces a significant inhibitory effect on enterprise participation willingness (β = −0.245, *p* < 0.001). This negative impact reveals enterprises’ complex trade-off process when evaluating UIC, consistent with Băban and Băban (2021) research findings on risk perception. Particularly, through control variable analysis, this research found that this inhibitory effect shows significant differences across enterprises of different sizes and ownership types, providing new empirical evidence for Neri et al.’s (2021) research on the moderating role of organizational characteristics in risk assessment.

The finding that policy support indirectly promotes participation willingness by enhancing enterprise self-efficacy (β = 0.298, *p* < 0.001) reveals an important mediating mechanism. This finding enriches Li et al.’s (2024) research on institutional environment impacts, while also deepening understanding of Yao et al.’s (2021) research on policy tool action mechanisms. Bootstrap mediating effect decomposition shows that policy support’s mediating effect through self-efficacy accounts for 42.3% of the total effect (indirect effect = 0.125), while direct effects account for 57.7% (direct effect = 0.173), suggesting policy design needs to balance capability building and immediate incentives. Research shows that policy support functions not only by directly reducing collaboration barriers but more importantly by enhancing enterprises’ capability perception, stimulating their endogenous motivation. The discovery of this indirect action mechanism provides new ideas for policy design.

Organizational characteristics as important moderating variables (β = 0.187, *p* < 0.01) significantly influence the relationship strength between various factors and participation willingness. This finding supports Mathisen and Jørgensen’s (2021) discussion on the importance of organizational characteristics in knowledge collaboration, while also deepening understanding of Cirella and Murphy’s (2022) research on the relationship between organizational capabilities and collaboration effectiveness. Through multi-group analysis, it was found that enterprises with stronger innovation capabilities and more abundant resources not only respond more positively to policy support but also demonstrate clear advantages in risk management and opportunity capture.

### 5.2 Theoretical contributions

The theoretical contributions of this research are reflected in multiple extensions to social cognitive theory, inter-organizational collaboration theory, and policy support theory. By integrating these theoretical perspectives, this research provides a more comprehensive framework for understanding the psychological pathways underlying enterprise participation in UIC than previously available in the literature. Regarding social cognitive theory, the research constructs an integrated framework explaining enterprise participation in UIC behavior by introducing cognitive factors such as observational learning, self-efficacy, and outcome expectations. This framework breaks through the limitations of existing research primarily relying on resource dependence theory and knowledge management theory, providing a new theoretical perspective for understanding inter-organizational collaboration.

In terms of inter-organizational collaboration theory, the research reveals the mediating mechanism through which policy support influences enterprise participation willingness via self-efficacy, deepening understanding of policy tool action mechanisms. In particular, the research finds that policy support not only directly influences enterprise participation decisions but, more importantly, forms sustained endogenous motivation by enhancing their capability perception. The revelation of this action mechanism provides a new analytical dimension for policy effect evaluation.

Regarding policy support theory, the research constructs a more complete theoretical model by introducing the moderating effect of organizational characteristics. Research results show that enterprises’ innovation capabilities and resource endowments not only directly influence their participation decisions but also significantly moderate their response level to external support. This finding enriches research on the role of organizational characteristics in policy effect transmission mechanisms, providing theoretical basis for enhancing policy precision.

### 5.3 Practical implications

The findings of this research have important implications for advancing UIC practices. Policy makers need to recognize that effective policy support is reflected not only in direct resource input but, more importantly, in stimulating enterprise endogenous motivation through capability building. It is suggested that local governments, in conjunction with industry associations, establish “UIC mentor databases,” matching university experts with enterprises by industry, providing technical diagnosis-project management-achievement transformation full-cycle guidance, and incorporating them into provincial-level science and technology plan assessment indicators. Based on enterprise heterogeneity characteristics found in this research (such as innovation capability and resource endowment differences), it is recommended to construct a tiered policy toolkit. For high innovation capability enterprises (such as large technology companies), policies should focus on improving intellectual property protection systems, providing R&D tax credits, and supporting their leadership in industry-university-research joint research projects; for small and medium enterprises, UIC special support funds need to be established, implementing “collaboration mentorship programs” (Mentorship Program), with universities or industry associations providing technical matching and management guidance, reducing their trial-and-error costs and risk perception. Additionally, regional UIC demonstration case libraries can be established, along with enterprise capability diagnosis and customized training, systematically enhancing enterprises’ collaboration confidence (self-efficacy) and project management capabilities.

Enterprise managers should recognize the opportunities and challenges brought by UIC, establishing systematic risk assessment and management mechanisms while focusing on expected benefits. Research findings show that observational learning is an important pathway to enhance participation confidence, and enterprises can optimize their collaboration strategies by deeply analyzing successful cases and drawing experience lessons. Enterprises can adopt benchmarking strategies, regularly visiting successful UIC enterprises (such as Huawei-university joint laboratories), and establishing internal UIC knowledge sharing platforms to reduce cognitive uncertainty. Meanwhile, enterprises should emphasize capability building, enhancing UIC project execution effectiveness by improving internal management mechanisms and cultivating professional teams.

As important UIC participants, universities need to strengthen understanding of enterprise needs and provide more targeted collaboration proposals. This requires universities to pay more attention to market demands in research orientation and talent cultivation, enhancing collaboration efficiency and achievement transformation capabilities by establishing professional UIC management teams. Particularly, universities should value differentiated collaboration strategies with different types of enterprises, providing more precise support for enterprises.

## 6 Conclusion and prospects

### 6.1 Conclusion

By constructing and validating an enterprise participation in UIC influence mechanism model based on social cognitive theory, this research systematically reveals the action mechanisms of factors such as observational learning, self-efficacy, and outcome expectations. Research shows that outcome expectations are the strongest direct influencing factor, while policy support indirectly promotes enterprise participation through enhancing self-efficacy. Organizational characteristics play an important moderating role in this process. These findings not only enrich UIC theoretical research but also provide important implications for policy making and enterprise practices. Future research directions include exploring differential characteristics of different types of UIC, as well as the impact of environmental dynamics on enterprise participation behavior. This research provides important theoretical and practical references for deepening understanding of UIC influence mechanisms, optimizing policy design, and enhancing collaboration effectiveness.

### 6.2 Limitations

This study has three primary limitations. First, the reliance on cross-sectional data restricts our ability to infer causal relationships; longitudinal designs could better capture the dynamic evolution of participation willingness. Second, while the sample includes enterprises from diverse industries in China’s coastal regions, findings may not generalize to inland provinces or other developing countries with distinct institutional environments. Third, the study does not differentiate between types of UIC (e.g., joint R&D versus talent cultivation), which may involve unique cognitive pathways. Future research could address these gaps by tracking enterprises over time and comparing mechanisms across regions and collaboration formats.

### 6.3 Future research directions

Three promising directions emerge from this study. First, investigating how digital transformation—such as AI-driven platforms—reshapes UIC participation through cognitive pathways could yield novel insights. Second, comparative analyses across industries (e.g., high-tech versus traditional manufacturing) may reveal sector-specific dynamics in how self-efficacy and policy support interact. Third, cross-national studies could explore how institutional maturity influences the effectiveness of policy tools, particularly in regions where UIC is still nascen. These efforts would deepen our understanding of UIC’s cognitive foundations and inform context-sensitive policy design.

## Data Availability

The original contributions presented in the study are included in the article/Supplementary material, further inquiries can be directed to the corresponding author.
